# In vitro and in vivo aphrodisiac properties of the seed extract from *Allium tuberosum* on corpus cavernosum smooth muscle relaxation and sexual behavior parameters in male Wistar rats

**DOI:** 10.1186/s12906-017-2008-5

**Published:** 2017-12-01

**Authors:** Xingli Tang, Opeyemi J. Olatunji, Yifeng Zhou, Xilin Hou

**Affiliations:** 1 College of Horticulture, Nanjing Agricultural University, Weigang No.1, Nanjing, Jiangsu 210095 China; 2 Faculty of Thai Traditional Medicine, Prince of Songkla University, Hat Yai, 90112 Thailand; 3 Institute of Botany, Jiangsu Province and Chinese Academy of Sciences, Nanjing Botanical Garden, Nanjing, 210014 China

**Keywords:** *Allium tuberosum*, Aphrodisiac, Sexual behavior, Potency

## Abstract

**Background:**

*Allium tuberosum* is a well-known spice as well as a herb in traditional Chinese medicine, used for increasing libido and treating erectile dysfunction. However, not many studies have been done to evaluate the sexual enhancing properties of *A. tuberosum*. The aim of this study was to evaluate the aphrodisiac and vasorelaxant properties of *A. tuberosum* on corpus cavernosum smooth muscle (CCSM) as well as checking the effect on enhancing male rat sexual behavior, libido, potency as well as its spermatogenic properties.

**Method:**

The seeds were powdered and sequentially extracted with hexane, ethyl acetate and butanol. Male Wistar rats were administered with graded doses of the *n*-BuOH extracts (ATB) of *A. tuberosum* (50, 100, 200 and 400 mg/kg) and Viagra was used as the positive control drug. The extract/drug was administered by gastric probe once daily for 45 days and the sexual behavior was analyzed by exposing the male rats to female rats in the estrus period.

**Results:**

ATB relaxed corpus cavernosum smooth muscle (68.9%) at a concentration of 200 μg/ml. The results obtained from the animal studies indicated that ATB significantly increased mount frequency (MF), intromission frequency (IF), ejaculation frequency (EF), ejaculation latency (EL) and markedly reduced post ejaculatory interval (PEI), mount latency (ML), and intromission latency (IL). Furthermore, a remarkable increase in the test for potency was observed as witnessed by marked increase in erections, quick flips, long flips and total reflex. In addition, ATB significantly improved the sperm viability and count as well as increased the concentrations of testosterone, follicle stimulating hormone (FSH), and phosphatases in the treated animals.

**Conclusion:**

Thus our results suggest that *A. tuberosum* could stimulate sexual arousal and enhance sexual execution in male rats, thus providing valuable experimental evidence that *A. tuberosum* possesses sexual enhancing properties.

## Background

Sexuality is a major fundamental principle of reproduction involving conjugation, conception and procreation. Sexual dysfunction is the inability to attain normal sexual activity, including loss or partial erection, inability to keep erection, premature ejaculation, reduced libido, orgasm, arousal disorder and lack of detumescence. Varying degrees of sexual dysfunction is estimated to occur in approximately 30 million men globally [1, 2]. Substances that have the ability to stimulate or increase sexual desire, performance, arousing sexual instinct, increase pleasure and enjoyment are referred to as aphrodisiacs. Some notable examples include Levodopa, Amyl nitrite and Viagra [3, 4]. Aphrodisiacs exerts their sexual enhancing effects by increasing the flow of blood, promoting erection and by causing relaxation of corpus cavernosal smooth muscle [5]. However, despite the increase in the use of some of the conventional aphrodisiacs for sexual dysfunction, medicinal plants have undoubtedly continued to gain attention as an alternative for the improvement or enhancement of sexual life. This is due to their accessibility, affordability and perceived to have fewer side effects.

The role of testicular phosphatases like acid phosphate (ACP) and alkaline phosphate (ALP) are considered vital as they are regarded as functional indicators of spermatogenesis. ACP are found in spermatogenic cells and the role of these enzymes are intensified during the differentiation of germ cells from spermatogonia into spermatocytes and spermatids. Lysosomal ACP is also involved in the digestion of phagocytosed exogenous compounds containing phosphate residues and they assist in the penetration of the spermatozoon through the egg. ALP on the other hand play a significant role as regards the motility and survival of sperm, testicular development as well as in the process of spermatogenesis [6, 7]. Alterations in the activities of these testicular phosphatases (ACP and ALP) could invariably result in damages to the seminiferous epithelium and loss of germinal elements. This results in the reduction of the production of sperm in the testes due to the decrease in the number of spermatids [8]. Therefore, maintaining normal activities of these enzymes is crucial for male sexuality.

Several varieties of plant species have been reported as stimulants of sexual activities. A few examples of these plants include *Allanblackia floribunda* Oliver, *Fadogia agrestis*, *Afromomum melegueta*, *Lepidium meyenii*, *Kaempferia parviflora*, *Terminalia catappa*, *Turnera diffusa*, *Cnestis ferruginea*, and *Allium tuberosum*, [2, 5, 9–11]. *Allium tuberosum* also known as Chinese chive is one of the species in the genus *Allium* with approximately 500 identified species [12]. Most of the species in this genus are used globally as food, spices and in herbal therapy. *A. tuberosum* is an edible green vegetable consumed in most part of China. Aside its use as food, the aerial part of the plant (leaves and seeds) are used in traditional Chinese medicine as a treatment for sexual enhancement, nocturnal emissions, abdominal pain, diarrhea, hematemesis, snakebite and asthma [13]. Recent pharmacological evaluations have indicated that the plant or its chemical components displays nematocidal, cytotoxic and antioxidant activities [14–17]. Chemical investigations of the different part of the plant revealed presence of steroidal saponins, alkaloids, phenylpropanoid glucoside, polysaccharides, flavonoids, sulphur containing compounds, minerals, nucleosides and amides from the plant [18–24].

Only two previous reports have described the efficacy of *A. tuberosum* in the improvement of male sexual behavior. Guohua et al. [25] reported the effect on male sexual behavior, while Sahoo et al. [26] used the plant in combination with seven other plants as a poly-herbal formulation in experimental model of male rats. However, no detailed mechanism of action of the claimed activity was investigated. In addition, the vascular activities of *A. tuberosum* have never been reported. The lack of penile erection may be partly due to the impairment in the relaxation of the smooth muscle which is related to the increased flow of blood to spaces of the corpus cavernosum. Even though the mechanism of action has not been fully elucidated, evidences suggests that nitric oxide is a primary mediator responsible for the relaxation in the corpus cavernosum [27, 28]. Thus this present study assesses the in vitro aphrodisiac properties of *A. tuberosum* by investigating the potential effect actions of the butanol extract of *A. tuberosum* on the relaxation corpus cavernosum smooth muscle as well as sexual behavior characteristics in normal male rats.

## Methods

### Chemicals

Estradiol benzoate and progesterone were procured from Sigma Chemical Co. (St. Louis, MO, USA). Viagra (sildenafil citrate) was purchased from Pfizer Inc. (Pfizer Inc., Pfizer Inc., Mission, KS, USA). All other chemicals used were of analytical grade.

### Plant material

*A. tuberosum* seed were purchased from Nanjing, Jiangsu province, China in April 2016. It was identified by Prof. Xilin Hou and a voucher specimen (AS. 100,101) was deposited at herbarium of Nanjing Agricultural University, Nanjing, China.

### Extraction of plant material

The seeds of *A. tuberosum* (3.0 kg) was powdered and successively extracted with 95% EtOH (1.5 L × 3) under soxhlet apparatus. The crude EtOH extract was filtered and concentrated under reduced pressure. The concentrate was re-suspended in water and successively extracted using hexane, EtOAc, and *n*-BuOH. The extracts were evaporated to dryness under reduced pressure. The *n*-BuOH extract was designated as ATB and was refrigerated and used for the chemical and bioactivity studies. ATB was used for this study based on previous reports on the presence of bioactive constituents in the butanol extracts of the plant [25, 29]. ATB was suspended in distilled water and used for the bioactivity study.

### Isolated rat CCSM study

The isolated rat CCSM study was conducted based on the previously described method [28]. Corpus carvernosal smooth muscle were dissected and mounted in an organ bath chamber containing modified Krebs-Henseleit physiological salt solution (PSS) solution having the stated composition: 7.11 g/l of NaCl, 0.36 g/l of KCl, 0.18 g/l of KH_2_PO_4_, 2.0 g/l of NaHCO_3_, 0.2 g/l of CaCl_2_, 0.4 g/l of MgSO_4_, 2.0 g/l of glucose and 0.01 g/l Na_2_EDTA. One end of the muscle was tied to a steel hook of the perfusion bath and the other end was attached to an isotonic force transducer for the measurement of isotonic tension. The changes observed in the isotonic tension were recorded on a computerized calibration program. The corpus cavernosum muscle was perfused with 2 ml Krebs-Henseleit-PSS solution and was aerated with 95% O_2_ and 5% CO_2_ for 10 min to stabilize the reading. It was thereafter perfused with 2 ml of CaCl_2_ (8.0 mg/ml) in Krebs-Henseleit-PSS solution for contraction of the muscle. ATB (10, 50, 100 and 200 μg/ml) and Viagra (10 μg/ml) were added by super fusing on the corpus cavernosum smooth muscle at 37 °C for 15 min.

### Animal experimental procedure

The animals were kept in clean cages in a well-ventilated housed conditions and were fed with standard animal feed and water ad libitum and were housed at a temperature of 23 ± 2 °C with a 12 h light and 12 h dark cycle and relative humidity of 45–55%. All animal experiments were conducted in accordance with the National Institute of Health guide for the care and use of laboratory animals (NIH Publication No. 80–23; revised 1978) and in accordance with the guidelines of the Animal Ethical Committee of Nanjing Agricultural University (approval number: SYXK(su)2017/0007).

### Animals groupings and ATB/drug administration

Healthy male Wistar rats (280.00 ± 5.00 g body weight) were used and female Wistar rats of (200.00 ± 5.00 g body weight) were used for the study. The animals were obtained from the Animal House facility of Nanjing Agricultural University. Female rats were made receptive and brought into estrous by administration of estrogen benzoate (10 mg/kg body weight) orally and progesterone (0.50 mg/kg body weight) subcutaneously 48 and 6 h respectively [26]. Animals were randomly divided into six groups (6 rats per group). Group 1 was the control and were administered with distilled water (vehicle); Group 2 was treated with 5 mg/kg of Viagra (positive control), 3, 4, 5 and 6 (ATB 50, ATB 100, ATB 200 and ATB 400) were treated with 50, 100, 200 and 400 mg/kg body weight of the butanol extract (ATB) of *A. tuberosum*, respectively. The rats were orally gavaged with the extract/drug once daily for 45 days using a metal oropharyngeal cannula.

### Sexual behavior study

The procedure used was based on previous report [27]. Female rat receptiveness was confirmed prior to the sexual activity test by exposing them to male rats and female rats that showed the maximum receptivity were chosen for the experimental procedure. The male rats were trained by exposing them to sexually receptive females once daily for 4 consecutive days before the experiment. The sexual activity test was performed on the 15th, 30th and 45th day after the onset of the extract/drug administration. The experiment was carried out under the same lighting conditions, time and in the same laboratory. The male and sexually receptive female rats were introduced into the mating cages in a 1:1 ratio. The test was terminated at any point when a male rat failed to show sexual interest. The sexual training for each male was done for 15 min per time until sexual behavior was achieved, at the notice of the sexual behavior the males were exposed to receptive females. All sexual behavior tests were observed for 4 h.

### Measurement of sexual parameters

The following sexual behavior parameters were analyzed; Mount Latency (ML, time from the introduction of the female till the first mount by the male with pelvic thrusting); Intromission Latency (IL, the time taken from the introduction of the female to the first mount and vaginal penetration); Ejaculation Latency (EL, the time interval between the first intromission and ejaculation); Mount frequency (MF, the number of mounts at a specified time period); Ejaculation frequency (EF, the number of ejaculations from the time of introduction of the female rats to the male within a given time frame), Post Ejaculatory Interval (PEI, the time taken from ejaculation until the next intromission) and penile licking (the number of time animals licked their penis).

### Test for penile reflexes

The test for penile reflexes was performed as previously described [28, 29]. Thirty minutes after drug administration, the rats were placed laying on their back in a glass cylinder. The penile sheath was pushed behind the glands and sustained in this position for 20 min. The frequencies of the Erections (E), Quick flips (QF), Long flips (LF) and Total penile reflexes (TPR; TPR = E + QF + LF) were analyzed. The test for penile reflexes were performed in three replicates.

### Determination of sperm viability, motility and count

On the 45th day after the experimental study, the animals were anesthetized by inhalation of isoflurane (4%) and euthanized by cervical dislocation. The assessment was performed based on previous report [30]. In brief, caudal epididymis of the rats was dissected and an incision of 1 mm was made to obtain the sperm fluid on a microscope slide. Afterwards, 3 drops of normal saline were added in order to mobilize the sperm cells. The computation of the motile spermatozoa per unit area was determined, the epididymis sperm motility was thus assessed. The sperm viability was also determined with the aid of Eosin/Nigrosin stain. The sperm count was analyzed by homogenizing the epididymis in 5 ml of normal saline and then counted using a haemocytometer.

### Estimation of testicular hormones

The homogenate from the testes was used for the estimation of testosterone, dihydrotestosterone (DHT) (Wuhan Fine Biological Technology, Wuhan, Hubei, China) and follicle-stimulating hormone (FSH) (ERBA Fertikit, Germany) immuno-enzymatic assay kit based on the manufacturer’s manuals.

### Estimation of biochemical parameters in testicular homogenate

Following anaesthetization, the testes were also immediately but diligently excised from the rats, cleaned and homogenized in ice-cold 0.25 M sucrose solution (1:5 *w*/*v*). The homogenates were centrifuged at 10000 g (4 °C, 10 min) and the resulting supernatant was stored (−20 °C) to ensure maximum release of the testicular fractions for the determination of biochemical parameters. Acid phosphatase (ACP) and alkaline phosphatase (ALP) levels were determined based on the manufacturers’ instructions for the kits (Randox Laboratories Ltd., Antrim, UK).

### Isolation and purification

The active *n*-BuOH extract (ATB, 215 g) was subjected a series of chromatographic techniques such as successive silica gel column chromatography, Sephadex LH-20 and semi preparative RP-HPLC to afford compounds 1–19.

### Statistical analysis

Data were expressed as means ± SD. Statistical analysis were performed using SPSS (version 16.0, SPSS Inc., Chicago, IL, USA), one-way analysis of variance (ANOVA) and followed by Tukey test Post hoc analysis. Differences were considered significant at *p* < 0.05.

## Results

### Effect of ATB on the on isolated CCSM

**Fig. 1:**
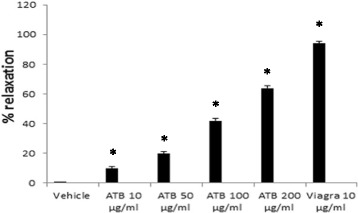
Effect of ATB and Viagra on isolated rat corpus cavernosum smooth muscle (CCSM). Data are expressed as mean ± SD (*n* = 6), one-way ANOVA followed by Tukey test Post hoc analysis. *P < 0.05 as compared to control group

As indicated in Fig. 1, ATB significantly relaxed the isolated rat CCSM in a concentration dependent manner. The highest dose of 200 μg/ml of ATB relaxed CCSM by 68.9%, while the reference drug Viagra had a relaxant effect of 100%.

### Effect of ATB on testicular hormone

The result obtained with respect to the effect of ATB on the level of hormones in the testis of the male rats are as indicated in Table 1. A marked increase was observed in the levels of testosterone, DHT and FSH in the testis of treat rats. The highest dose of ATB (400 mg/kg) had a 2-fold increase in the levels of the hormones when compared with the untreated control group.

**Table 1:** Effect of ATB on the level of testicular hormones in the male rats

Groups	Testosterone (nmol/L)	FSH (IU/L)	DHT (nmol/L)
Control	23.62 ± 0.58	2.14 ± 0.05	2.42 ± 0.05
Viagra	70.11 ± 0.45*	6.17 ± 0.07*	8.24 ± 0.10*
ATB 50	33.18 ± 0.39	2.61 ± 0.03	3.63 ± 0.03
ATB 100	42.46 ± 0.44*	3.04 ± 0.08*	4.18 ± 0.05*
ATB 200	46.20 ± 0.32*	3.38 ± 0.06*	4.38 ± 0.04*
ATB 400	60.34 ± 0.61*	4.21 ± 0.04*	6.01 ± 0.09*

Data are expressed as mean ± SD (*n* = 6). *P < 0.05 as compared to control group

### Effect of ATB on sexual parameters in normal male rats

**Fig. 2:**
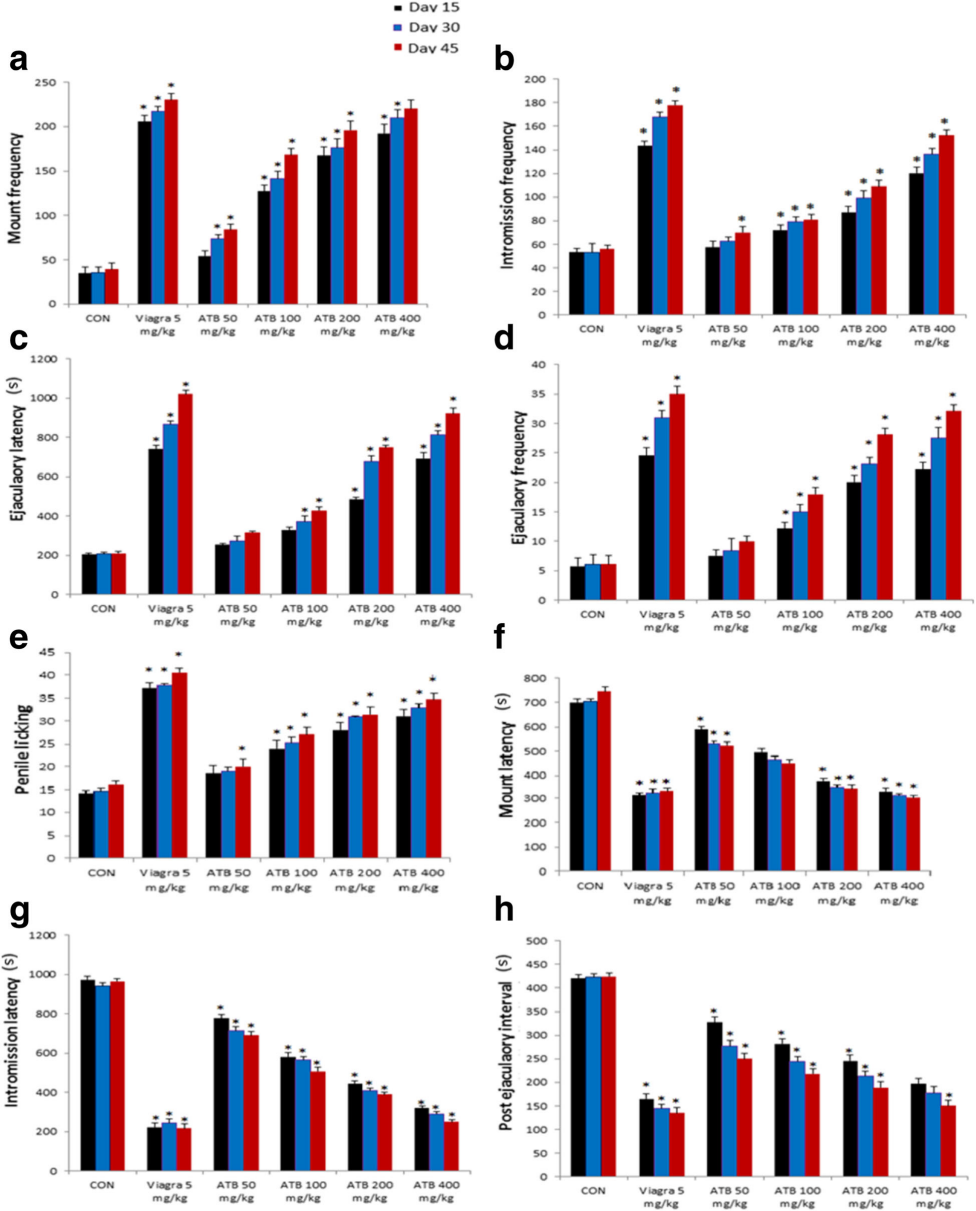
Effect of ATB and Viagra on sexual behavior of rats; (**a**) Mount frequency, (**b**) Intromission frequency, (**c**) Ejaculaotry latency, (**c**) Ejaculatory frequency, (**d**) penile licking, (**f**) Mount latency, (**d**) Intromission latency and (**f**) Post ejaculatory interval. Data are expressed as mean ± SD (*n* = 6), one-way ANOVA followed by Tukey test Post hoc analysis. *P < 0.05 as compared to control group

The female rats displayed proceptivity as observed by their ear-wiggling, lordosis and hopping while the treated male rats showed signs of enhanced sexual activity towards the female rats as witnessed by their eager and quick movement towards the female rats as well as some distinctly visible signs of pre-copulatory actions such as body-sniffing, anogenital exploration, circling around, which finally culminated in mounting [31–34]. The result obtained from the analysis of the various parameters relating to male rat sexual characteristics indicated that ATB significantly increased EL, MF, EF and IF in a dose dependent manner when compared with the rats in the untreated control group (Fig. 2a-d). The observed activity was in a dose dependent manner. In addition, ATB also led to a marked decrease in the ML, IL and PEI in comparison to the rats untreated control rats (Fig. 2f-h). The decrease in these parameters was observed in all administered doses of ATB.

### Effect of ATB on the rat penile potency

The test for potency as indicated in Table 2 shows that ATB at 400 mg/kg significantly increased the frequency of erections, long flips, quick flip and total penile reflex. The effect was relatively comparable with the Viagra group. Similarly, the other administered doses also had positive effect on penile potency in a dose dependent manner.

**Table 2:** Effect of ATB on penile reflexes in treated male rats

Parameter	Groups	Day 15	Day 30	Day 45
Erection	Control	6.21 ± 1.28	6.84 ± 1.48	6.98 ± 1.08
	Viagra	21.63 ± 1.18*	22.20 ± 1.39*	24.18 ± 1.56*
	ATB 50	8.91 ± 1.29	9.69 ± 1.44	10.37 ± 1.79
	ATB 100	10.77 ± 1.38	11.83 ± 1.29	13.37 ± 1.44
	ATB 200	12.06 ± 1.76*	13.78 ± 1.88*	15.22 ± 1.72*
	ATB 400	17.70 ± 1.58*	19.89 ± 1.71*	22.04 ± 2.18*
Quick flip	Control	4.11 ± 0.82	4.77 ± 1.01	5.32 ± 0.65
	Viagra	14.66 ± 2.01*	16.27 ± 1.78*	20.44 ± 1.99*
	ATB 50	5.29 ± 1.27	5.85 ± 1.11	9.34 ± 2.06
	ATB 100	7.01 ± 1.13	8.77 ± 1.36	11.88 ± 1.40
	ATB 200	9.61 ± 1.66*	12.53 ± 1.80*	13.76 ± 1.51*
	ATB 400	13.11 ± 1.09*	15.86 ± 1.88*	17.55 ± 1.48*
Long flip	Control	2.99 ± 0.77	3.47 ± 0.94	5.06 ± 1.10
	Viagra	15.49 ± 1.72*	20.11 ± 1.52*	22.30 ± 2.06*
	ATB 50	4.01 ± 0.96	5.39 ± 1.14	7.22 ± 1.06
	ATB 100	6.93 ± 1.46*	8.20 ± 1.54*	10.28 ± 1.47*
	ATB 200	9.43 ± 0.89*	10.52 ± 1.60*	12.10 ± 1.22*
	ATB 400	12.32 ± 1.85*	15.04 ± 1.64*	18.21 ± 2.22*
Total reflex	Control	13.31 ± 1.82	14.72 ± 2.07	17.36 ± 2.32
	Viagra	51.78 ± 2.66*	58.58 ± 3.11*	66.92 ± 3.14*
	ATB 50	18.21 ± 1.92	20.93 ± 2.32	26.93 ± 2.83
	ATB 100	24.71 ± 2.29*	28.80 ± 2.83*	35.53 ± 3.36*
	ATB 200	31.10 ± 2.28*	36.83 ± 2.12*	21.08 ± 3.24*
	ATB 400	43.13 ± 2.86*	50.49 ± 3.38*	57.80 ± 3.91*

Data are expressed as mean ± SD (n = 6). *P < 0.05 as compared to control group

### Effect of ATB on sperm motility, count and viability

As shown in Table 3, ATB treatment led to a significant increase in sperm motility, sperm count and sperm viability of the treated rats as compared to the untreated control group of rats.

**Table 3:** Effect of ATB on sperm motility, count and viability

Groups	% Motility	Count (Mio/ml)	% Viability
Control	52.86	50.68 ± 0.09	60.06
Viagra	69.09*	68.17 ± 0.07*	66.80
ATB 50	56.22	58.11 ± 0.15	63.78
ATB 100	63.28*	66.21 ± 0.12*	68.40*
ATB 200	68.71*	72.04 ± 0.17*	72.94*
ATB 400	79.24*	84.42 ± 0.21*	77.38*

Data are expressed as mean ± SD (n = 6). *P < 0.05 as compared to control group

### Effect of ATB on the activities of ALP and ACP in the testis

The activities of ALP and ACP enzymes in the testis were observed to be significantly increased in ATB treated rats in comparison with the untreated control group. Interestingly, ATB at 200 and 400 mg/kg had a better effect on the activities of these enzymes than the positive control drug which was not significantly different from the untreated control (Table 4).

**Table 4:** Effect of ATB on testicular levels of ALP and ACP enzymes

Groups	ALP (U/I)	ACP (U/I)
Control	6.85 ± 0.11	4.37 ± 0.08
Viagra	7.33 ± 0.12	5.28 ± 0.07
ATB 50	7.14 ± 0.08	4.86 ± 0.14
ATB 100	8.03 ± 0.10*	5.54 ± 0.21*
ATB 200	8.22 ± 0.07*	7.38 ± 0.08*
ATB 400	9.94 ± 0.15*	7.80 ± 0.13*

Data are expressed as mean ± SD (*n* = 6). *P < 0.05 as compared to control group

### Isolation and characterization of isolated compounds from ATB

**Fig. 3:**
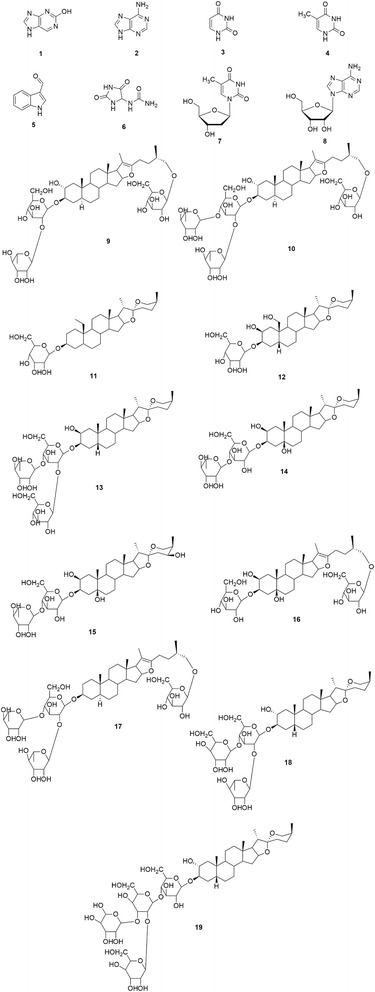
Chemical structures of compounds 1–19 isolated from the seed of *A. tuberosum* (ATB)

An extensive chemical isolation and chemical purification of ATB extract using various chromatographic techniques led to the isolation of 19 compounds (Fig. 3). The compounds were identified as 2-hydroxy-purine (**1**), adenine (**2**) [35], uracil (**3**), thymine (**4**) [36], 3-formylindole (**5**), allantoin (**6**), thymidine (**7**) [37], adenosine (**8**), tuberosides A (**9**) and B (**10**) [38], tuberosine A (**11**) and B (**12**) [18], tuberosides N (**13**), P (**14**), Q (**15**), R (**16**) and T (**17**), [Sang et al., [39] neogitogenin 3-*O*-α-_L_-rhamnopyranosyl-(1--- 2)-*O*-[β-_D_-glucopyranosyl-(1---4)]-β-_D_-galactopyranoside (**18**), gitogenin 3-*O*-{O-β-_D_-glucopyranosyl- (1---2)-*O*-[β-_D_-xylopyranosyl-(1---3)]-*O*-β-_D_-glucopyranosyl-(1---4)-β-_D_-galactopyranoside (**19**) [Mimaki et al., [40]; Kim et al., [41], using spectroscopic analyses (NMR, MS and IR spectroscopy), as well as comparison of their spectra with previous reports. This is the first report on the isolation of compounds **18** and **19** from *A. tuberosum*.

## Discussion

Erectile dysfunction (ED) is a health condition which leads to the inability of a man to maintain an erection of his penis sufficiently for a period of time for normal sexual intercourse or for achieving sexual satisfaction. The currently available treatment for ED have several unpleasant side effects as well as limited efficiency [42]. Therefore, alternative treatment which are safer and shows more efficacy is required and these can be assessed from the use of herbal/medicinal plants which have inherent aphrodisiac properties. Several medicinal plants have been used as medicines for combating menace of sexual dysfunction. Notably among these plants is *A. tuberosum,* a widely consumed vegetable in China with renal protective activity as well as for the treatment of impotence and nocturnal emissions [22]. This present study indicated that *A. tuberosum* treatment enhanced stimulatory effects on male sexual behavior. Indices of male sexual efficiency such as mount and intromission frequencies are parameters that are usually measured in the determination sexual motivation, penile erection efficiency and ejaculatory reflexes activation [3, 43]. The increase in the frequencies of mount and intromission may be indicative of the ability of the drug to control erectile dysfunction and asexual arousal disorders. It was observed that the treatment doses of ATB extract increased mounts and intromissions efficiency in all treated rats. These effects were observed to be significantly elevated and comparable to the positive control drug in the treated dose of 400 mg/kg. Furthermore, the marked increase observed in the ejaculation latency of administered rats implies that ATB could extend the copulation period. These results are thus suggestive of the improved sexual vigour, potency, motivation and copulatory performance in the treated rats, suggesting the positive impact of *A. tuberosum* on vital indicators of sexual vigour and potency thus enhancing sexual performance.

It is generally accepted that mount latency (ML) and intromission latency (IL) are parameters that are considered to be a mark of sexual motivation. These parameters are inversely proportional to sexual carving. An increase in these parameters suggests lack or loss of sexual appetite/drive [3]. Treatment of rats with ATB extract was associated with a decline in mount latency and intromission latency, thus signifying improved sexual stimulation and motivation in the rats. Post ejaculatory interval (PEI) measures the recovery rate from exhaustion after the first set of mating, as well as a measure for potency and sexual drive [44]. The decreased rate of post ejaculatory interval in groups administered with ATB thus indicates that the extract may have positive effect on enhancing potency and libido, which could suggest that *A. tuberosum* may facilitate erectile function and the highest adminstered dose could produce similar effect as viagra in the male rat sexual parameters. In addition, the effect of ATB extract on potency was also investigated. The effect of ATB on the frequencies of erections (E), quick flips (QF) and long flips (LF). ATB cause a marked increase in the tested parameters of penile reflexes (E, QF and LF) in all the treated rat groups when compared to control group.

Previous studies have suggested that the level of testosterone in the blood is essential for arousing sexual desire or stimulation. This sex hormone plays important modulatory role of male sexual behavior in the CNS. It is also noteworthy to indicate that the activity of these sex hormones has a direct correlation with erectile function in the peripheral nervous system (PNS) [45]. The Leydig cells found in the testes is responsible for producing testosterone via a process called steroidogenesis [46]. High levels of testosterone observed in the treated rats treated with ATB revealed the ability of the fraction to stimulate the production of testosterone itself. Our results indicated that *A. tuberosum* extracts could stimulate increase in the level of testosterone thus improving sexual motivation and activity of the rats. The relaxation of the smooth muscle has been proposed to be associated with erection [47]. Nitric oxide that is released by the endothelium of the arteries that supply the penis, corpus cavernosum is responsible for mediating the relaxation of corpus cavernosum smooth muscle via the formation of guanosine 3′5′ cyclic monophosphate [48]. Furthermore, it has been hypothesized that plants which show relaxation effects on corpus carvenosum smooth muscle can increase or promote blood flow and or retention of blood in the penile organ which is a vital requirement for penile erection and copulation [45]. This hypothesis seems valid as there is a strong correlation between sexual activity and the relaxation of corpus carvenosum smooth muscle.

The effect of plant bioactives on sexual enhancement is thought to be mediated through different mechanisms of action such as induction of the relaxation of smooth muscle corpus cavernosum through the L-arginine/nitric oxide pathway [49], enhancement of acetylcholine and transmural nerve stimulation, which in turn activates relaxation. A number of previous reports have also linked the sexual enhancing capabilities of these bioactives in enhancing the level of sex hormones and /or triggering the action of sex hormones in the body. In particular, previous literatures have reported that plant saponins have the ability to serve as a precursor for sexual hormones like testosterone as well as increase the levels of reproductive hormones such as follicle stimulating hormone and luteinizing hormone [50, 51]. Saponins also have the ability to act on neurotransmitters such as nitric oxide resulting in the relaxation of the corpus cavernosal smooth muscle [49, 51, 52]. Based on these it could be inferred that the major chemical constituents in *A. tuberosum* which are steroidal saponins and nucleosides may have played a critical role in the aphrodisiac activity. These constituents present in ATB extract may have caused a stimulation of the body’s testosterone levels which in turn is expressed in the enhancement of the male rat’s sexual capacity as indicated in this study. Saponins have been previously suggested to have the ability to bind to certain hormone receptors/enzymes involved in the synthesis of these hormones, thus enhancing the physiological function of the hormone [53].

Spermatogenesis, the process that produces spermatozoa from stem cells of the spermatogonia. The number of sperm in the testes is increased during this process. The results from this present study indicated a significant dose dependent increase in the sperm count, sperm motility and sperm viability of the rats administered with ATB suggesting the possibility of the spermatogenic properties of the extract. The effect of plant or its bioactive on the spermatozoa characteristics is thought to be mediated through the hypothalamic-pituitary-testicular axis of the animal. The increase in sperm count and motility in the treated rats might due to the effect of the extract on the maturation of the spermatozoa as well as the androgen enhancing effect of ATB. The extract may also have facilitated the spermatogenesis as well as aiding the functioning of epididymis [54].

Alkaline phosphatase (ALP) is involved in the mitosis of spermatogenic cells as well as glucose transport that is necessary by the spermatozoa in the seminal fluid and for steroidogenesis. Acid phosphatase (ACP) are found in the in lysosome of leydig cells in the testis and are involved in the synthesis of protein by abduction of sex hormones and sperm physiology [55]. Alterations in the activities of these enzymes are indicative of spermatogenesis function [56, 57]. There was an increase in the activities of ALP and ACP in the testis of ATB treated rats thus suggesting the possibility of the extract in enhancement of steroidogenesis.

## Conclusion

Taken together, our findings clearly demonstrate that the butanol extract from *A. tuberosum* significantly enhances sexual behavior parameters, thus indicating that the plant possesses potent aphrodisiac activity.

## Abbreviations

*A. tuberosum*: *Allium tuberosum*
ACP: Acid phosphate
ALP: Alkaline phosphate
ATB: *n*-butanol extracts of *Allium tuberosum*
CCSM: Corpus cavernosum smooth muscle
DHT: Dihydrotestosterone
E: Erection
EF: Ejaculation frequency
EL: Ejaculation latency
EtOAc: Ethyl acetate
EtOH: ethanol
FSH: Follicle stimulating hormone
IF: Intromission frequency
IL: Intromission latency
LF: Long flips
MF: Mount frequency
ML: Mount latency
*n*-BuOH: *n*-butanol
PEI: Post ejaculatory interval
PSS: Physiological salt solution
QF: Quick flips
TPR: Total penile reflexes
